# Household air pollution and under-five mortality in India (1992–2006)

**DOI:** 10.1186/s12940-016-0138-8

**Published:** 2016-04-26

**Authors:** Sabrina Naz, Andrew Page, Kingsley Emwinyore Agho

**Affiliations:** Centre of Health Research, School of Medicine, Western Sydney University, Building 3, Campbelltown Campus, Locked Bag 1797, Penrith, NSW 2571 Australia; School of Science and Health, Western Sydney University, Campbelltown Campus, Locked Bag 1797, Penrith, NSW 2571 Australia

**Keywords:** Household air pollution, Under-five mortality, Child mortality, Cooking fuel, India

## Abstract

**Background:**

Household air pollution (HAP) - predominantly from cooking fuel is a major public health hazard and one of the leading causes of respiratory illness and deaths among children under-five years in India. This study investigates the association between HAP from cooking fuel and under-five mortality using India’s National Family and Health Survey (NFHS) datasets over the period 1992–2006 (total of 166,382 children), and the extent to which the association differed by environmental and behavioral factors affecting level of exposure.

**Methods:**

The association between HAP and under-five mortality of three age-groups (neonatal age between 0–28 days, post-neonatal age between 1–11 months and children aged between 12–59 months) was examined using multi-level logistic regression models.

**Results:**

HAP was associated with mortality among children aged under-five (OR = 1.30, 95%CI = 1.18-1.43, *P* < 0.001) and was more strongly associated in sub-group analyses of post-neonatal mortality (OR = 1.42, 95%CI = 1.19-1.71, *P* < 0.001) and child mortality (OR = 1.42, 95%CI = 1.05-1.91, *P* = 0.021) than neonatal mortality (OR = 1.23, 95%CI = 1.09-1.39, *P* = 0.001). The association was stronger for households in rural areas and for households without a separate kitchen using polluting fuel, and in women who had never breastfed for all age-groups.

**Conclusion:**

Use of cooking fuel in the household is associated with increased risk of mortality in children aged under-five years. Factors relating to access to clean fuels, improvements in infrastructure and household design and behavioral factors are discussed, and can result in further declines in under-five mortality in India.

## Background

India is the second most populous and seventh largest country by area in the world located in South Asia, and currently is one of the ten fastest growing economies in the world [1]. In the last five decades in India, there has been extensive improvement in poverty reduction, literacy, health standards and human development, however, there remains significant population challenges in relating to health and sanitation [1]. More than 90 % of the rural population and 31 % of the urban population in India still rely primarily on solid fuels as a domestic source of energy [1–3]. Household air pollution (HAP) from solid fuels (such as wood, animal dung, crop residues, charcoal and coal) for cooking and heating is a substantial cause of respiratory illness and death, due to a range health damaging pollutants such as fine particles, carbon monoxide (CO_2)_, nitrogen oxides (NO_2)_, sulphur dioxide (SO_2),_ benzene, butadiene, formaldehyde, polyaromatic hydro-carbons and a number of other chemicals, [4, 5] and remains a major public health concern in the developing world [6, 7]. A recent study from India indicated that 56 % of children aged under-five remained with their mother at all times during cooking [2], and that proximity to smoke from solid fuel use is associated with an increase in the risk of health problems among young children [8, 9].

According to World Health Organization (WHO), 3.5 % of the total burden of disease in India country has been attributable to HAP [10] and a previous study from India has indicated that solid fuel use was responsible for 20 % of deaths among children <5 years [11, 12]. In addition, 24 % of total deaths among children under five in India was associated with acute respiratory illness (ARIs) [12, 13] which has also been identified as the leading cause of death of children less than five by the 2010 Global Burden of disease (GBD) study [14]. The under-five mortality in India declined from 125 per 1,000 live births in 1990 to 74.6 per 1,000 live births in 2005–06 [15], and despite projections that it will further decline to 70 per 1,000 live births by 2015, this still does not achieve the Millennium Development Goal 4 (MDG4) target of 42 per 1,000 live births to reduce mortality among children under five by two-thirds [15].

A number of previous studies in India have reported the effects of HAP on respiratory diseases among young children [12, 16–20] and associations between HAP and under-five mortality with other health outcomes (e.g., low birth weight, respiratory illnesses among young children) [11, 21–23]. However, those studies have been limited to surveys of limited geographic areas, or hospital based data sources for specific regional populations or focused on all types of HAP (for example, including tobacco use), and not exclusively cooking fuel [24–29]. To date, no studies in India have examined changes in the association between HAP and under-five mortality over time, or investigated the role of environmental and behavioural factors that might affect the level of exposure to HAP (for example, place of residence, location of kitchen, and breastfeeding status). Accordingly, the objective of this study was to investigate trends in the association between HAP from cooking fuel and under-five mortality for three consecutive age groups (neonatal, post-neonatal and child), and to assess how this is affected by key environmental and behavioral factors using large-scale nationally representative data over the period 1992–2006.

## Methods

### Data sources

The data in this study were extracted from India’s National Family and Health Survey (NFHS) datasets for the years 1992–93 (NFHS-1), 1998–99 (NFHS-2) and 2005–06 (NFHS-3). The NFHS are nationwide surveys based on a representative sample of households throughout the country under the authority of the Ministry of Health and Family Welfare (MOHFW), Government of India, and implemented by the International Institute for Population Sciences (IIPS), Mumbai with technical assistance from Macro International of Calverton, Maryland, USA, as a part of its Demographic and Health Surveys Program [1, 30, 31]. To date, the three NFHS surveys (NFHS-1, NFHS-2 & NFHS-3) have collected demographic and health data by interviewing ever-married women (aged 15–49 years) and men (aged 15–54 years) using a stratified sample of households based on a two-stage cluster design [1, 30, 31]. NFHS-3 covered all 29 states of India, which includes more than 99 % of India’s population [1].

A total of 303,361 ever-married women of reproductive age (112,357 from urban and 191,009 from rural areas) were included in the three datasets with a response rate of 95.4 % in women across the three datasets (NFHS-1, NFHS-2 and NFHS-3). This study was based on information relating to 166,382 singleton live-born children, of whom 11,311 died in the 5-years prior to the survey. An index period of five years was to minimize recall bias of child birth and death information self-reported by the mother.

### Study outcomes

The analysis for under-five mortality was carried out for three successive age groups: neonatal, post-neonatal and child mortality, using the following definitions:

- *Neonatal mortality:* The number of deaths during the first 28 days of life (0–28 days). Defined as, Number of neonatal deaths/Total number of live births
- *Post-neonatal mortality:* The number of deaths between one month and the first birthday (1–11 months). Defined as, Number of post-neonatal deaths/Total number of live births
- *Child mortality:* The number of deaths between exact ages one and five (12–59 months). Defined as, Number of child deaths/Total number of live births

The outcome variables were considered dichotomous for the analysis, where age at death was either yes (=1) denoting death occurred during these any three periods of age or no (=0) denoting the child survived during the age-period.

### Exposure to cooking fuel

The main exposure variable was type of cooking fuel used in the household. The respondents were asked, “What type of fuel does your household mainly use for cooking?” and in response 12 types of cooking fuel were reported. In the analysis, these fuels were grouped into two categories on the basis of exposure to cooking smoke: “clean fuels” (electricity, liquid petroleum gas (LPG), natural gas and biogas) and “polluting fuels” (kerosene, coal/lignite, charcoal, wood, straw/shrubs/grass, agricultural crop waste and dung cakes). NFHS analyses have previously classified cooking fuel as “solid” and “non-solid” fuels, where kerosene was categorised in the non-exposed (i.e. “clean fuel”) group [1, 30, 31]. However, some previous studies have reported kerosene as a polluting fuel and have found significant associations between under-five mortality or respiratory illness among children and kerosene fuel use [21, 32, 33]. For this reason, kerosene was categorised in the polluting fuels group.

### Potential confounders

Place of residence (categorized as “urban” or “rural”), household wealth index (categorized as “high income”, “middle income” or “low income”), mother’s education (categorized as “secondary or higher”, “primary” or “no education”), mother’s working status (categorized as “working” or “not working”) and type of house (categorized as “pucca”, “semi-pucca” or “kachha”) were included as markers of socio-economic status, and have previously been identified as potential confounders of the association between HAP and under-five mortality [11, 21–23, 33–38]. The household wealth index was constructed using principal components analysis, with weights for the wealth index calculated by giving scores to the asset variables such as ownership of transport, durable goods and facilities in the household [1, 30, 31, 39]. “Low income” referred to the bottom 40 % of households, “middle income” referred to the middle 40 % of households, and “high income” referred to the top 20 % of households, based on the approach described by Filmer and Pritchett [39]. Mother’s age (categorized as <20, 20–29, 30–39 and 40–49 years) and sex of the child (categorized as “female” or “male”) were also considered as potential confounders of the association between HAP and under-five mortality.

Breastfeeding status of mother (categorized as ever breastfed “yes” or “no”) and location of kitchen (categorized as separate room used as kitchen “yes” or “no”) were also considered *a priori* factors that may indicate different levels of exposure to polluting fuels. No separate kitchen used for cooking in the household as an indicator of proximity to polluting fuel use has also been presented to be an significant factor associated with high exposure to HAP [24, 26–28, 36, 37, 40]. Additionally, breastfeeding has been shown to be a protective factor for under-five mortality, generally in neonatal and infancy period [33, 34, 41–44] which may reduce the greater risk of exposure associated with HAP. Hence, analyses sought to determine whether the magnitude of the association between HAP and under-five mortality differed by past breastfeeding status.

### Statistical Analysis

The association between type of cooking fuels and under-five mortality was investigated using a series of multilevel logistic regression models adjusted for the potential confounders of household wealth, place of residence, mother’s age, mother’s education, mother’s working status, sex of child, breastfeeding status, kitchen location and type of house. Changes in neonatal, post neonatal and child mortality incidences from HAP over time were also investigated using a trend analysis across 1992–93, 1998–99 and 2005–06 NFHS data by specifying ‘period’ as a continuous variable. To identify the overall effect of HAP from cooking fuels with neonatal, post-neonatal and child mortality, pooled analyses were also conducted. The extent of divergence or convergence between the slopes of period specific trends within each variable over the study period (1992–2006) was assessed by testing the interaction between period and a given confounding variable using likelihood ratio tests.

Stratified analyses were also conducted by breastfeeding status and by location of kitchen to determine whether the magnitude of the effect of the exposure on outcomes differed across levels of these variables. Breastfeeding status (ever breastfed “yes” or “no”) and location of kitchen (separate room used as kitchen “yes” or “no”) were each also combined with type of cooking fuel as composite ordinal variables to investigate different level of exposure to HAP for under-five mortality outcomes.

The “Svy” command was used for calculating weighted cumulative incidence estimates of mortality to adjust for the cluster sampling survey design. Random effects multilevel logistic regression models were conducted by using the “xtlogit” command and for likelihood ratio test for interaction “lrtest” command was used. Adjusted risk differences were also estimated from logistic regression model using the “margins” command. All analyses were carried out in STATA version 13.1 (Stata Corp: College Station, TX, USA).

### Ethics

The Demography and Health Survey (DHS) project sought and obtained the required ethical approvals from ethics committees in India before the surveys were conducted. Informed consent was obtained from study participants before their participation in the surveys. Publicly available, de-identified datasets were used in this study following approval from The DHS Program.

## Results

**Fig. 1:**
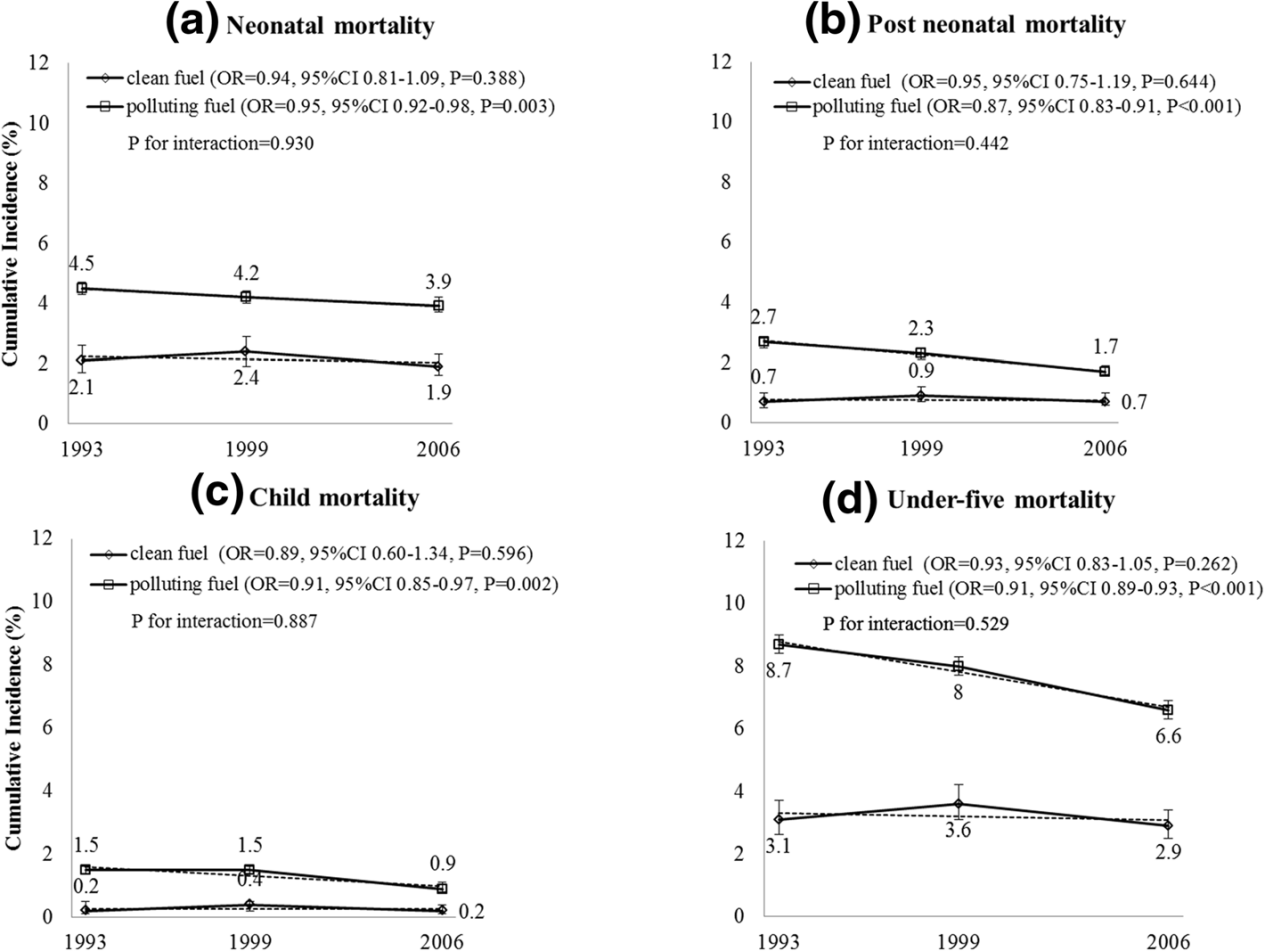
Mortality trend in all age-groups of children under-five associated with HAP in India. Note: P for interaction based on likelihood ratio test of ‘period’ by ‘exposure to polluting fuels’. Odds ratios (OR) for linear trend (i.e. the period effects) for each exposure group (clean or polluting fuel) were adjusted for wealth index, place of residence, mother’s age, mother’s education, mother’s working status, sex of child, breastfeeding status, kitchen location and type of house

**Fig. 2:**
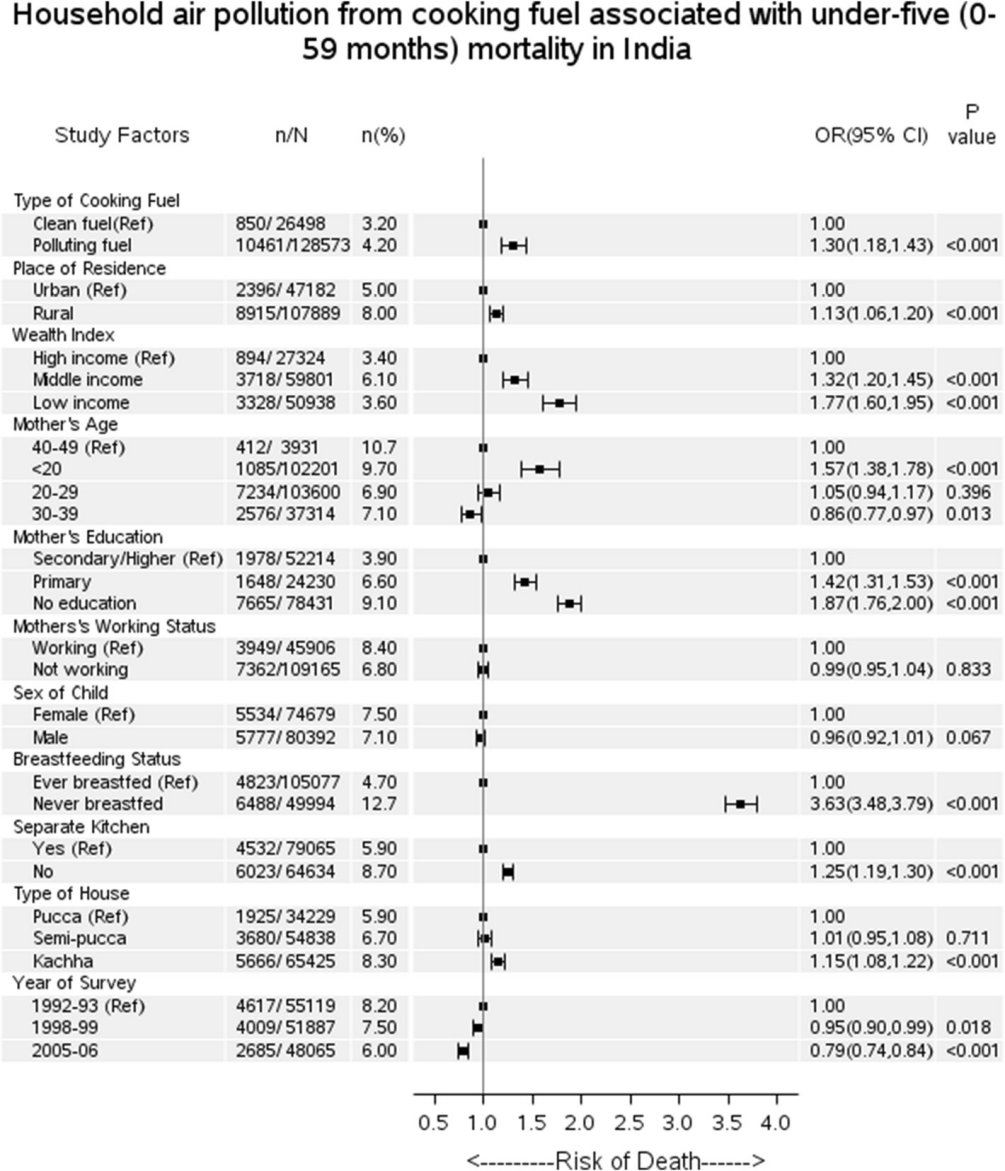
HAP associated with overall under-five mortality in India: a pooled analysis for 1992–2006

The overall under-five mortality incidence proportion in India decreased from 8.7 % per year in 1992 to 6.6 % per year in 2006 for those using polluting fuels for cooking. Decreasing trends were also evident for each age group, where the neonatal mortality incidence proportion declined from 4.5 % in 1992 to 3.9 % in 2006, post-neonatal mortality incidence proportion from 2.7 % in 1992 to 1.7 % in 2006 and child mortality incidence proportion from 1.5 % in 1992 to 0.9 % in 2006 (Fig. 1). Use of polluting fuels (kerosene, coal/lignite, charcoal, wood, straw/shrubs/grass, agricultural crop waste and dung cakes) for cooking was associated with a higher risk of post-neonatal (OR = 1.42, 95%CI = 1.19-1.71, *P* < 0.001) and child mortality (OR = 1.42, 95%CI = 1.05-1.91, *P* = 0.021) than neonatal mortality (OR = 1.23, 95%CI = 1.09-1.39, *P* = 0.001) after adjusting for household wealth, place of residence, mother’s age, mother’s education, mother’s working status, sex of child, breastfeeding status, kitchen location and type of house (Table 1). Use of polluting fuels and under-five mortality showed statistically significant association (OR = 1.30, 95%CI = 1.18-1.43, *P* < 0.001) after adjusting for confounders (Fig. 2). Corresponding risk differences between use of clean fuel and polluting fuel were found (0.68 %, 95%CI = 0.33 %-1.03 %) for neonatal, (0.61 %, 95%CI = 0.35 %-0.87 %) for post-neonatal, (0.34 %, 95%CI = 0.28 %-0.40 %) for child mortality and (1.50 %, 95%CI = 1.01 %-1.99 %) for overall under-five mortality.

**Table 1:** HAP associated with neonatal, post neonatal and child mortality in India: a pooled analysis for 1992–2006

	Neonatal				Post neonatal				Child			
Study factors	n^e^	n (%)^f^	OR^b^ 95%CI	*P* value	n^e^	n (%)^f^	OR^b^ 95%CI	*P* value	n^e^	n (%)^f^	OR^b^ 95%CI	P value
Type of Cooking Fuel												
Clean fuel^a, c^	565	2.1	1.00		216	0.8	1.00		69	0.3	1.00	
Polluting fuel^d^	5517	4.2	1.23 (1.09-1.39)	0.001	3138	2.3	1.42 (1.19-1.71)	<0.001	1806	1.3	1.42 (1.05-1.91)	0.021
Place of Residence												
Urban^a^	1366	2.9	1.00		723	1.4	1.00		307	0.7	1.00	
Rural	4716	4.3	1.15 (1.06-1.24)	0.001	2631	2.3	1.05 (0.94-1.16)	0.383	1568	1.4	1.16 (0.99-1.35)	0.054
Wealth Index												
High income^a^	587	2.2	1.00		231	0.9	1.00		76	0.3	1.00	
Middle income	2111	3.6	1.27 (1.13-1.42)	<0.001	1100	1.7	1.37 (1.15-1.63)	<0.001	507	0.8	1.51 (1.13-2.02)	0.005
Low income	3328	4.7	1.58 (1.39-1.79)	<0.001	1980	2.6	1.78 (1.48-2.14)	<0.001	1271	1.7	2.37 (1.76-3.18)	<0.001
Mother's Age												
40-49^a^	187	4.9	1.00		135	3.4	1.00		90	2.3	1.00	
<20	702	6.4	2.25 (1.88-2.68)	<0.001	308	2.6	1.37 (1.11-1.71)	0.004	75	0.7	0.43 (0.32-0.60)	<0.001
20-29	3921	3.8	1.22 (1.04-1.43)	0.013	2144	2.0	0.98 (0.81-1.18)	0.814	1169	1.1	0.80 (0.64-1.01)	0.052
30-39	1268	3.5	0.93 (0.79-1.09)	0.404	767	2.1	0.81 (067–0.99)	0.038	541	1.6	0.86 (0.68-1.08)	0.192
Mother's Education												
Secondary/Higher^a^	1301	2.6	1.00		499	0.9	1.00		178	0.3	1.00	
Primary	944	3.9	1.28 (1.16-1.41)	<0.001	505	1.9	1.66 (1.44-1.91)	<0.001	199	0.8	1.56 (1.25-1.94)	<0.001
No education	3825	4.7	1.52 (1.40-1.65)	<0.001	2344	2.7	2.17 (1.92-2.45)	<0.001	1496	1.7	2.67 (2.22-3.22)	<0.001
Mother's Working Status												
Working^a^	2035	4.4	1.00		1145	2.3	1.00		769	1.6	1.00	
Not working	4047	3.8	1.02 (0.97-1.09)	0.424	2209	2.0	1.05 (0.97-1.13)	0.233	1106	1.0	0.85 (0.77-0.93)	0.001
Sex of Child												
Female^a^	2705	3.7	1.00		1721	2.3	1.00		1108	1.5	1.00	
Male	3377	4.2	1.17 (1.10-1.23)	<0.001	1633	1.9	0.87 (0.81-0.93)	<0.001	767	0.9	0.64 (0.58-0.71)	<0.001
Breastfeeding Status												
Ever breastfed^a^	2488	2.5	1.00		1389	1.3	1.00		946	0.9	1.00	
Never breastfed	3594	7.2	3.64 (3.44-3.86)	<0.001	1965	3.7	3.59 (3.33-3.87)	<0.001	929	1.8	2.34 (2.13-2.58)	<0.001
Separate Kitchen												
Yes^a^	2601	3.4	1.00		1321	1.7	1.00		610	0.8	1.00	
No	3043	4.5	1.16 (1.09-1.22)	<0.001	1827	2.6	1.24 (1.15-1.34)	<0.001	1153	1.6	1.45 (1.30-1.61)	<0.001
Type of House												
Pucca^a^	1104	3.5	1.00		557	1.6	1.00		264	0.8	1.00	
Semi-pucca	1975	3.7	0.98 (0.91-1.07)	0.687	1661	2.0	1.04 (0.93-1.17)	0.454	583	1.1	0.99 (0.84-1.16)	0.874
Kachha	2985	4.4	1.16 (1.09-1.22)	0.003	1122	2.4	1.08 (0.97-1.21)	0.148	1020	1.5	1.20 (1.03-1.41)	0.017
Year of Survey												
1992-1993^a^	2391	4.3	1.00		1438	2.5	1.00		788	1.4	1.00	
1998-1999	2104	4.0	0.95 (0.89-1.02)	0.137	742	2.1	0.88 (0.81-0.95)	0.002	731	1.4	1.08 (0.97-1.20)	0.161
2005-2006	1587	3.6	0.88 (0.81-0.95)	0.002	1174	1.6	0.74 (0.67-0.82)	<0.001	356	0.8	0.68 (0.58-0.79)	<0.001

Note: ^a^Reference category, ^b^odds ratio adjusted for wealth index, place of residence, mother’s age, mother’s education, mother’s working status, sex of child, breastfeeding status, kitchen location, type of house and year of survey, ^c^clean fuels: electricity, LPG, natural gas, biogas, ^d^Polluting fuels: kerosene, coal/lignite, charcoal, wood, straw/shrubs/grass, agricultural crop and animal dung, ^e^n = number of mortality cases of children for neonatal, post neonatal and child age-group, ^f^percentage of mortality cases

Stratified analyses to examine different levels of exposure to HAP showed more than 5-fold greater risk of mortality in women who never breastfeed and who used polluting fuels for cooking (compared to breastfeeding women who used clean fuels), with robust associations evident for child (OR = 10.47 95%CI = 7.13-15.37, *P* < 0.001) and post-neonatal (OR = 8.87 95%CI = 6.94-11.33, *P* < 0.001) mortality than neonatal (OR = 5.36 95%CI = 4.65-6.19, *P* < 0.001) mortality (Table 2). In addition, the risk of under-five mortality was also higher for the women who ever breastfed but used polluting fuel for cooking (Table 2).

**Table 2:** Risk of mortality by breastfeeding status and kitchen location

	Neonatal				Post neonatal				Child			
Study Factors	n^e^	n (%)^f^	OR^b^ 95 %(CI)	*P* value	n^e^	n (%)^f^	OR^b^ 95 %(CI)	*P* value	n^e^	n (%)^f^	OR^b^ 95 %(CI)	*P* value
Combined Association of Breastfeeding Status and Use of Cooking fuel												
Ever breastfed & used clean fuels^a, c^	203	1.5	1.00		67	0.6	1.00		27	0.2	1.00	
Ever breastfed & used polluting fuels^d^	2285	2.6	1.58 (1.37-1.83)	<0.001	1322	1.4	2.76 (2.16-3.53)	<0.001	919	1.0	4.72 (3.22-6.92)	<0.001
Never breastfed & used clean fuels^c^	362	2.7	1.73 (1.46-2.06)	<0.001	149	1.0	2.15 (1.61-2.87)	<0.001	42	0.3	1.49 (0.92-2.41)	0.109
Never breastfed & used polluting fuels^d^	3232	8.2	5.36 (4.65-6.19)	<0.001	1816	4.4	8.87 (6.94-11.33)	<0.001	887	2.2	10.47 (7.13-15.37)	<0.001
*Linear trend*			*1.83 (1.78-1.88)*	*<0.001*			*1.84 (1.78-1.91)*	*<0.001*			*1.57 (1.50-1.64)*	*<0.001*
Combined Association of Kitchen Location and Use of Cooking fuel												
Separate kitchen used clean fuels^a, c^	419	2.1	1.00		146	0.7	1.00		51	0.3	1.00	
Separate kitchen used polluting fuels^d^	2182	3.8	1.83 (1.65-2.04)	<0.001	1175	1.9	2.81 (2.36-3.34)	<0.001	559	0.9	3.79 (2.84-5.05)	<0.001
No separate kitchen used clean fuels^c^	131	2.3	1.30 (1.06-1.58)	0.010	66	1.3	1.88 (1.40-2.51)	<0.001	17	0.2	1.38 (0.80-2.39)	0.249
No separate kitchen used polluting fuels^d^	2912	4.6	2.34 (2.11-2.59)	<0.001	1761	2.6	4.03 (3.40-4.78)	<0.001	1136	1.7	7.37 (5.56-9.76)	<0.001
*Linear trend*			*1.21 (1.18-1.24)*	*<0.001*			*1.32 (1.28-1.37)*	*<0.001*			*1.54 (1.47-1.61)*	*<0.001*

Note: ^a^Reference category, ^b^odds ratio, ^c^clean fuels: electricity, LPG, natural gas, biogas, ^d^Polluting fuels: kerosene, coal/lignite, charcoal, wood, straw/shrubs/grass, agricultural crop and animal dung, ^e^n = number of mortality cases of children for neonatal, post-neonatal and child age-group, ^f^percentage of mortality cases

Analyses combining location of kitchen (separate room used as kitchen or not) and use of cooking fuels, showed strong evidence of an association between households using polluting fuels and no separate kitchen (compared to households with a separate kitchen who used clean fuel for cooking), with stronger associations for child mortality (OR = 7.37, 95%CI = 5.56-9.76, *P* < 0.001) than post-neonatal (OR = 4.03, 95%CI = 3.40-4.78, *P* < 0.001) and neonatal mortality (OR = 2.34, 95%CI = 2.11-2.59, *P* < 0.001) (Table 2). There was also an indication of association between use of polluting fuels in a separate kitchen and neonatal, post-neonatal and child mortality (Table 2). A sensitivity analysis was conducted including kerosene as a “clean fuel” group which did not change results substantially (only 6.94 % of women reported the use of kerosene for cooking in three survey years) (data not shown).

## Discussion

Under-five mortality in India has declined substantially over time, however, the association between household use of polluting fuels for cooking and under-five mortality has not changed significantly over time. The risk of death was higher in post-neonatal and child age groups than the neonatal age-group, and were generally consistent with previous studies in India and Nigeria [11, 21–23, 34]. Findings are also consistent with proposed biological mechanisms relating to key pollutants such as fine particles, carbon monoxide (CO) and a number of other chemicals present in solid cooking fuels compared to clean cooking fuels. Under-five mortality in all consecutive age groups was higher in rural areas compared to urban and in households without a separate kitchen (compared to separate kitchen) for cooking their meals. There was also a lower risk of mortality in mothers who had ever breastfed compared to never breastfed which was consistent with its previously reported role in respiratory outcomes [41, 43, 44] and which appeared to attenuate the effects of polluting fuel use, with a higher risk of under-five mortality among never breastfeeding (compared to ever breastfeeding) mothers using polluting cooking fuels.

This study found rural children were at greater risk of death than children from rural areas which was consistent with previous studies in India [11, 21, 22]. Approximately 90 % of rural households in India depend on solid fuels as a domestic source of energy and the only option for cooking, whereas cleaner fuels (natural gas/LPG) were more common in urban areas [1]. Other socioeconomic factors associated factors (for example, kachha (poor) house, mother’s with no education, and lower income) are also likely influences of the use of polluting fuels in rural areas.

The location of kitchen has previously been shown to be an important factor in studies of HAP and under-five mortality in developing country settings (such as, African countries and Bangladesh) [36, 37], and are also consistent with observed associations between HAP and under-five mortality in the present study which have not been shown before for India using nationally representative data sources. Analyses that incorporated the location of kitchen found a higher risk of neonatal, post-neonatal and child mortality when mothers reported no separate kitchen and used polluting fuels for cooking. Moreover, this study found that households using polluting fuels in indoor kitchen without partitions were at higher risk of death of their children under-five than household with separate kitchen both in urban and rural areas. Findings suggest that a separate kitchen in the household could reduce the death of young children even where polluting fuels were used for cooking. This is because households without a separate kitchen have higher concentrations of particles and children mainly less than five year of age were exposed to higher level of smoke as they spent many hours indoors [45].

Breastfeeding has previously been shown to protect infants against infection and has been reported as a protective factor for reducing risk of respiratory illness among infants [41, 43], and thus was a behaviour that was investigated to determine whether breastfeeding status might attenuate the association between HAP and under-five mortality. Analyses showed substantial differences in the association between HAP and under-five mortality between women who did or did not breastfeed. The strong effect of the breastfeeding status of mother was examined in the neonatal and post-neonatal period compared to older children who reported using polluting fuels, but who also breastfed their children. Therefore, breastfeeding in the first one year of life was found to be a protective factor for lower risk of mortality among young children associated with HAP, consistent with previous similar studies in Nigeria [34], but not previously shown in a South Asian context.

There are a number of methodological considerations to be taken into account when interpreting these findings. This study was based on a series of cross-sectional secondary datasets, with a number of potential sources of bias including selection, misclassification and recall bias. Firstly, the classification of cooking fuel may be a source of misclassification bias, as some households use a combination of polluting and clean fuels. Moreover, the DHS survey only collected information of primary fuel use, and there was no data for secondary fuel use. For example, one recent study on India indicated that dwellings reporting kerosene as their primary fuel frequently shift to cooking with biomass fuel (such as wood) which may cause higher concentrations of HAP [21, 46], and attenuate associations between HAP and under-five mortality. In addition, this study did not account for past exposure to HAP or recent changes in cooking methods because of its cross-sectional design.

Secondly, information on birth and death of children was self-reported by mothers, which may be a source of recall bias. The present study constrained analyses to those children born within a five year period prior to the survey date in order to minimise the likelihood of recall bias (and maximise the study sample size). Thirdly, this study used cross-sectional data for analysis, and it is difficult to clearly define temporal relationships between the exposure and outcome when collected at the same point of time.

Furthermore, we considered all-cause mortality for our analysis of the association between HAP and under-five mortality. Cause of death information was not available in the NFHS dataset used for this study. Verbal autopsy questionnaires are employed for identifying cause of death in developing countries, including India; however this information is often of variable quality and was not collected for India NFHS data. Not only pneumonia and acute respiratory infections but also other factors, such as preterm birth complications, low birth weight, nutritional conditions and diarrhoea also affect mortality among under-five children but we could not measure cause-specific death due to lack of data. The 2010 GBD study indicated that acute lower respiratory infections was the second leading cause of death (after preterm birth complications) in under-five children in India [14]. HAP from cooking fuel is a primary cause for respiratory infections among under-five children and thus can be associated with the deaths caused by those illnesses. However, including all-cause mortality will also include mortality outcomes not associated with HAP, which is likely to be a source of ascertainment bias in the outcome, and lead to an underestimation of the association between HAP and the cause-specific outcomes noted above.

This study also did not measure actual levels and patterns of exposure to emission from cooking smoke due to the absence of such information in DHS data. Proxy environmental and behavioural measures were defined to examine the effects of level of exposure. Location of kitchen was used as a proxy measure to investigate the effects of different level of exposure associated with HAP and under-five mortality and several studies in India noted a significant association between HAP and kitchen location [18, 45, 47, 48]. Other proxy measures such as 'presence of window', and 'cooking under a chimney' were available at NFHS-3; however this information were not collected in previous surveys.

Despite these methodological concerns, this present study used large-scale nationally representative DHS data with a very high response rate of 95.4 %, and was the first study to pool datasets over a long period (1992–2006), a period in India characterised by rapid socio-economic development. No previous studies have investigated trends and differentials in the association between HAP and under-five mortality in India over this long period, or assessed the role of environmental and behavioural factors that may be points of intervention and health promotion at the national level in the Indian context to further contribute to declines in under-five mortality in the post-MDG period.

The magnitude of the association between HAP and under-five mortality remained consistent over time in all age-groups and higher exposure to cooking fuel can substantially increase the risk of respiratory illness and child deaths in developing countries like India. According to NFHS-3, only 0.4 % of the households had access to electricity and 24.7 % of households used LPG/natural gas, with the remainder still relying on polluting fuels in both urban and rural areas [1]. Despite the potentially small relative risk of under-five mortality associated with HAP, it remains a common exposure in the population and therefore the population attributable risk - of this preventable risk factor - remains a public health priority for India.

Raising awareness about the health risk related with HAP and the use of polluting fuel is needed in rural and low income urban areas. Radio and television have been identified as useful media to raise awareness and to reach poorer sub-groups of the population [49, 50]. The most important intervention to reduce exposure to polluting fuel is ready access to clean fuels such as LPG/natural gas, biogas and electricity [18, 19, 22]. Many countries like Brazil, Bolivia, Ecuador have decreased exposure to pollution from cooking fuel by promoting liquid fuel supported by government policy [22, 51]. Switching to clean fuel is advisable, however, it is not an affordable option for many poor families in India and supplying cleaner fuel to rural household is expensive, and thus requires long term intervention because of poor infrastructure in India [18, 19, 22].

Behavioral change interventions also have potential, and are shorter term alternatives in India to reduce child exposure from HAP [18, 19, 22]. Studies from India suggested that improvements in natural household ventilation, particularly windows and improved stoves (stoves with chimney) might lead to a reduction in HAP [18, 19, 22]. Cooking stoves with a chimney also have health advantages, via reductions in carbon monoxide emissions and incidence of respiratory infections [52, 53]. Studies showed that India has distributed ten millions of improved cooking stoves since 1980 and plans to distribute 150 million in the next decade [54, 55]. Findings from this study suggests that, behavioral interventions are likely to play a key role in decreasing childhood deaths in India by promoting the use of improved cooking stoves, raising awareness for cooking in a separate kitchen and removing children from the cooking area while cooking, increasing natural ventilation in household and implementing concerted health promotion campaigns to inform people about public health hazard relating to HAP.

## Conclusions

The overall under-five mortality rates in India have decreased substantially over the study period (1992–2006). While HAP was associated with a modest increase in risk of mortality in children under five, the ubiquitous use of polluting fuel in India and associated population attributable risk, confirms that HAP remains an important public health problem. Cooking fuel is a modifiable risk factor that can be changed by improvements in house design, health system policies, infrastructure, behavioural intervention and economic development of the country.

## Abbreviations

HAP: Household Air Pollution
NFHS: National Family and Health Survey
WHO: World Health Organization
ARI: Acute Respiratory Infection
GBD: Global Burden of Disease
MDG: Millennium Development Goal
MOHFW: Ministry of Health and Family Welfare
IIPS: International Institute for Population Sciences
LPG: Liquid Petroleum Gas
DHS: Demography and Health Survey
